# Characterization, Expression Profile Analysis, and Functional Prediction of *UGP* Gene Family in *Dendrocalamus brandisii*

**DOI:** 10.3390/plants14101458

**Published:** 2025-05-14

**Authors:** He Li, Chongyang Wu, Xiangyi Li, Junlei Xu, Zhanchao Cheng, Jian Gao

**Affiliations:** Key Laboratory of National Forestry and Grassland Administration on Bamboo & Rattan Science and Technology, International Center for Bamboo and Rattan, Beijing 100102, China; lihe@icbr.ac.cn (H.L.); wcy@icbr.ac.cn (C.W.); lixiangyi@caf.ac.cn (X.L.); xjl@icbr.ac.cn (J.X.); czc@icbr.ac.cn (Z.C.)

**Keywords:** *Dendrocalamus brandisii*, *DbUGP*, gene family evolution, duplication events, protein structure, expression analysis

## Abstract

UDP-glucose pyrophosphorylase (UGPase) is essential for carbohydrate metabolism, catalyzing UDP-glucose synthesis, a precursor for sucrose and cellulose biosynthesis. While *UGP* genes have been widely studied in plants, their functions in *Dendrocalamus brandisii* remain unclear. This study identified and characterized the *DbUGP* gene family using the whole genome and transcriptome data of *D. brandisii*, in conjunction with whole genome data from 10 additional species through sequence alignment, phylogenetic analysis, gene structure and motif exploration, protein structure prediction, and expression profiling. Phylogenetic analysis showed eight identified *DbUGPs* clustered with two *OsUGPs* in two clades. Gene structure, motif, and collinearity analyses indicate conservation with other bamboo *UGPs*. The gene family exhibited segmental duplications. Expression profiling revealed *DbUGP1*/*5* were highly expressed in flowers, while others were enriched in shoots, buds, and culms. *DbUGP1*/*4*/*8* were significantly downregulated during culm maturation. Protein structure prediction indicated two conformations with catalytic sites in disordered coil regions. WGCNA identified co-expression modules and protein interaction networks centered on *DbUGP1*/*4*, while KEGG enrichment indicated their functions in metabolism, signal transduction, and stress adaptation. Promoter analysis identified cis-regulatory elements responsive to light, MeJA, and ABA. This study suggests that the evolutionarily conserved *DbUGPs* exhibit mutual coordination and dynamic expression during *D. brandisii* growth, providing fresh insights into their functional roles.

## 1. Introduction

UDP-glucose (UDP-Glc) serves as a precursor for the synthesis of many important products in carbohydrate metabolism, including sucrose and cellulose [1]. UDP-glucose pyrophosphorylase (UGPase), which catalyzes the formation of UDP-Glc, is centrally located within this biosynthetic pathway [2]. As a key enzyme, *the UGP* gene plays a crucial role in the synthesis of carbohydrate compounds. The *UGP* gene, encoding UGPase, is widely distributed across various tissues and organs in plants, participating in numerous stages of plant growth and reproduction. However, its expression levels exhibit variation. For instance, *PtUGP1* shows broad expression across all tissues in poplar, while *PtUGP2* is predominantly expressed in flowers and roots [3]. Similarly, *GhUGP* expression can be detected in the roots, stems, leaves, and flowers of cotton, with the highest expression observed in the floral organs [4]. In *Oryza sativa*, the *OsUGP1* and *OsUGP2* genes are expressed in the roots, flowers, and leaves, although the expression levels of the two genes differ significantly, with *OsUGP1* showing particularly strong expression in the floral organs [5]. In *Arabidopsis thaliana*, tissue-specific expression patterns of *UGP* genes reveal that *AtUGP1* and *AtUGP2* are expressed in all organs, particularly in flower buds and flowers. Further GUS fusion staining indicates that the deepest coloration is found in the *A. thaliana* microspores, pollen, and stigma, suggesting higher expression levels of the target genes. Notably, *AtUGP1* is expressed at higher levels than *AtUGP2*, implying that it may play a dominant role in the development of *A. thaliana* floral organs [6]. This suggests that there may be functional divergence or gene redundancy among *UGP* family members.

**Figure 1:**
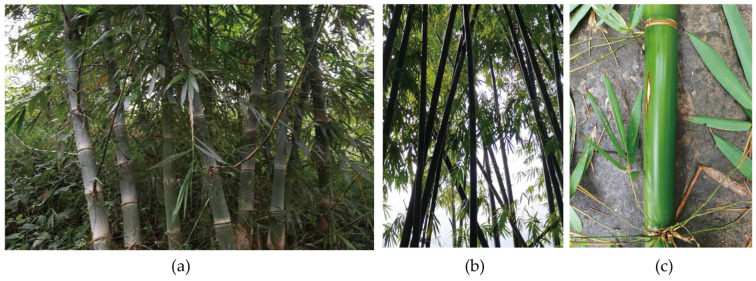
The growth condition of *D. brandisii* and the morphology of its culms. (**a**) Base part of culms; (**b**) Middle part of the culms; (**c**) Stable internode of culm (the 15th internode from the base).

Despite the central role of the *UGP* gene family in carbohydrate metabolism, research on this gene family in *D. brandisii* remains scarce. This gap in research may be attributed to the large and difficult-to-annotate genomes of bamboo species, as well as the complex and enigmatic characteristics of their growth and development. These include notable features such as rapid growth [7], energy supply for underground rhizomes and above-ground sheaths [8,9], highly developed secondary cell wall synthesis capabilities, developmental patterns from base to apex [10,11], and irregular flowering cycles [12]. *D. brandisii* is a large, evergreen clumping bamboo species native to tropical and subtropical regions, belonging to the Poaceae family and the Bambusoideae subfamily (Figure 1). It originates from the southern and northeastern regions of India, as well as Myanmar, and is primarily distributed in southern China, South Asia, and Southeast Asian countries [13,14]. Previous chloroplast genome analyses have indicated that *D. brandisii* is closely related to *Dendrocalamus latiflorus*. *D. brandisii*, *Dendrocalamus hamiltonii*, and *Dendrocalamus asper* are the three main sweet bamboo species found in China, South Asia, and Southeast Asia, respectively. These species are known for their high-quality fresh bamboo shoots with a sweet taste. Among the bamboo species commonly consumed in China, *D. brandisii* contains the highest levels of glutamate and sugars [15]. *D. brandisii’s* shoot has superior taste quality compared to *D. latiflorus* and even *Phyllostachys edulis* [16]. As a polyploid species, it grows larger and has harder wood than lower-ploidy bamboo species upon maturation. Given bamboo’s advanced secondary wall synthesis abilities and rapid growth, how does it effectively synthesize and utilize carbohydrate resources? Furthermore, bamboo’s material properties depend on the accumulation of cell wall fiber components, with cellulose distribution often higher than that of most herbaceous plants. This could be due to differential expression levels of genes involved in cellulose biosynthesis, leading to changes in the number and types of key enzymes involved.

Currently, research on the function of plant *UGP* genes has laid a solid foundation. These genes are enriched in lignin degradation metabolism and polysaccharide biosynthesis processes, such as cellulose synthesis [17,18]. The functional conservation of *UGP* genes across various plant species suggests that the role of *UGP* may be well-preserved in plant evolution [3]. Our team has obtained high-precision genomic data for *D. brandisii* [19]. This study identifies and characterizes the *UGP* genes in *D. brandisii* through bioinformatic analyses and differential gene expression profiling at various developmental stages. We have preliminarily inferred the potential molecular functions of the *DbUGPs*. This research lays a foundation for understanding the molecular mechanisms of key genes like *UGP* in the carbohydrate metabolism of *D. brandisii* and offers new insights into the broader biological functions of *UGP*.

## 2. Results

### 2.1. Identification of UGP Gene Families in D. brandisii

In this study, four, two, and two *UGP* gene family members were identified in the A, B, and C subchromosome groups of *D. brandisii*, respectively. These genes were confirmed through NCBI and SMART databases to contain the conserved UDP-glucose pyrophosphorylase (UDPGP) domain (Pfam: PF01704), a hallmark of this gene family. Based on their chromosomal positions, they were renamed as *DbUGP1* through *DbUGP8* (Table S1). The proteins encoded by these genes varied in size from 467 to 552 amino acids, with theoretical molecular weights ranging from 51,600.18 to 60,820.73 Da and isoelectric points between 5.28 and 6.39. Most of these proteins were predicted to exhibit high thermal stability and structural integrity. Subcellular localization predictions indicated that *DbUGPs* were localized in the cytoplasm. Transmembrane domain prediction indicated that none of the eight members possessed transmembrane regions. Furthermore, signal peptide analysis revealed that none of these proteins contained signal peptide sequences.

### 2.2. DbUGPs Were Evolutionarily Conserved and Distributed Across Two Clades with OsUGPs

To investigate the protein sequences of the identified *D. brandisii UGP* families, they were aligned with the UGP-A protein sequences from *A. thaliana* and *O. sativa* using NCBI BLASTp (Version 2.15.0). Results showed no significant differences in homology between the two AtUGP proteins and the eight *D. brandisii* UGP proteins. However, OsUGP2 demonstrated significantly higher homology with DbUGP1 and DbUGP5 (94.7–88%, approximately 7% higher), while OsUGP1 showed significantly higher homology with the remaining six *D. brandisii* UGP proteins (95.5–87.4%, approximately 8% higher).

**Figure 2:**
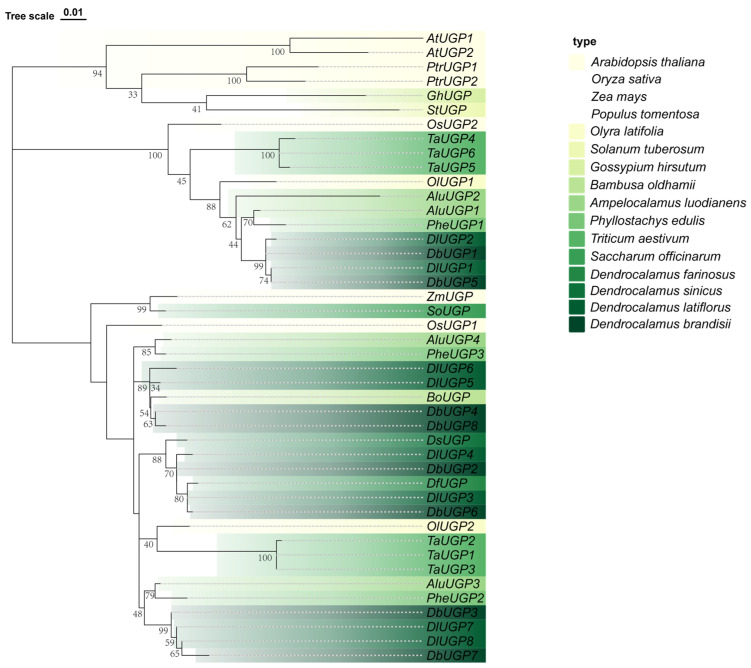
Phylogenetic tree of the *UGP* gene family. In the figure, gradient colors represent different plant categories: model plants (*A. thaliana*, *O. sativa*, *Z. mays*, *P. tomentosa*), *O. latifolia*, *S. tuberosum*, *G. hirsutum*, *B. oldhamii*, *A. luodianensis*, *P. edulis*, *T. aestivum*, *S. officinarum*, *D. sinicus*, *D. latiflorus, D. farinosus,* and *D. brandisii*.

To explore the evolutionary characteristics of the *UGP* gene family in *D. brandisii*, we employed the same methodology to identify members of the *UGP* gene family in several commonly studied species of Poaceae with complete genome data, including *D. latiflorus*, *P. edulis*, *Ampelocalamus luodianensis*, *Olyra latifolia,* and *Triticum aestivum*, as well as in model plants such as *A. thaliana*, *O. sativa*, *Zea mays*, *Populus tomentosa*, *Solanum tuberosum*, and the dicotyledonous plant *Gossypium hirsutum*. Additionally, we compiled and analyzed *UGP* genes from related species such as *Bambusa oldhamii*, *Dendrocalamus sinicus*, *Dendrocalamus farinosus,* and *Saccharum officinarum*, previously reported in the literature. A phylogenetic tree was subsequently constructed using Chiplot, encompassing 4 dicotyledonous plants and 12 monocotyledonous plants (including 8 species from the *Bambusoideae* subfamily) [20] (Figure 2). The phylogenetic analysis revealed three major clades. As an outgroup, the dicotyledonous species *P. tomentosa*, *G. hirsutum*, *S. tuberosum*, and *A. thaliana* clustered separately into a distinct phylogenetic clade, highlighting their evolutionary divergence from the monocot *UGP* genes. The eight *D. brandisii UGP* genes and other *UGP* genes from bamboo species clustered with the two *O. sativa UGP* genes in two distinct clades, demonstrating closer phylogenetic relationships to *O. sativa*. Consistent with the NCBI BLASTp (Version 2.15.0) results, *DbUGP1* and *DbUGP5* clustered with *OsUGP2*, while the remaining six *DbUGPs* clustered with *OsUGP1*. Similarly, in the hexaploid bamboo species *D. latiflorus*, more than half of its *UGPs* clustered with *OsUGP1*. Additionally, the *UGP* gene family members of *O. latifolia*, *A. luodianensis*, and *T. aestivum* were evenly distributed across two distinct phylogenetic clades.

### 2.3. Gene Conserved Motif, Structure Analysis, and Protein Functional Domain Prediction of DbUGPs

**Figure 3:**
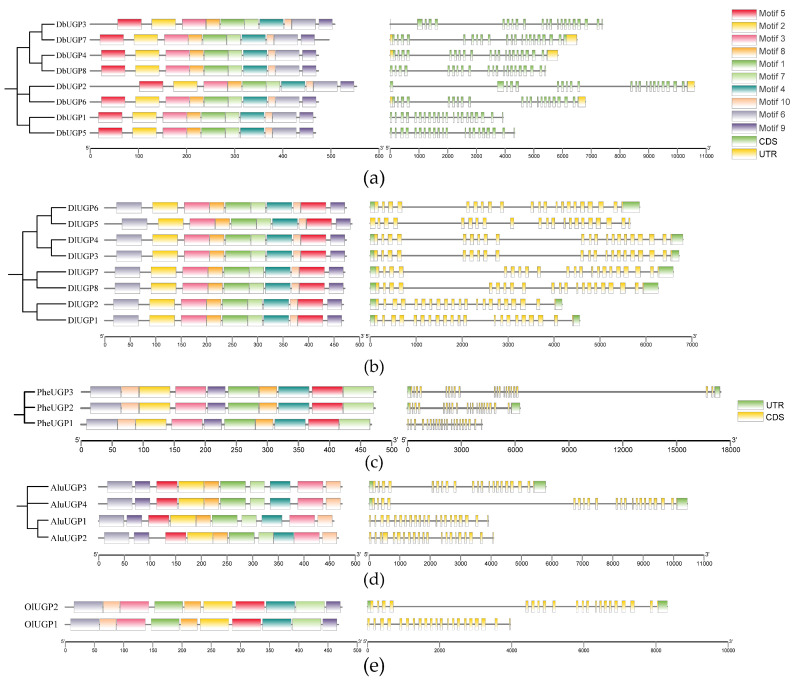
Phylogenetic tree, conserved motifs, and gene structure analysis of the *UGP* gene family in five bamboo species. (**a**) *D. brandisii*; (**b**) *D. latiflorusi*; (**c**) *P. edulis*; (**d**) *A. luodianensis*; (**e**) *O. latifolia*. Ten motifs are represented by different color blocks; in the schematic diagram of the gene structure, the green blocks represent CDS and the yellow blocks represent UTR in *D. brandisii*, whereas in the other bamboo species, the colors are reversed.

Conserved motifs are typically associated with protein functions. To identify the characteristic motifs of *UGPs*, 10 conserved motifs in the UGP proteins were identified using MEME software (Version 5.5.7) (Figure 3). These motifs were named Motif 1–Motif 10, and they were highly conserved across UGP proteins in each bamboo species. Notably, the relationship between *D. latiflorus* and *D. brandisii* was a close relationship, and all their protein sequences contain the absolutely conserved Motif 1–Motif 2, Motif 4–Motif 7, and Motif 10. These motifs’ sequence information was submitted to the PFAM database for functional queries (Table S2), indicating that most of the motifs were associated with the UDP-alpha-D-glucose (GO:0006011) metabolic process and exhibited uridylyltransferase activity (GO:0070569), particularly involving UTP: glucose-1-phosphate uridylyltransferase activity (GO:0003983). This suggests that these motifs play a key role in catalyzing the reaction of UTP with glucose-1-phosphate to generate UDP-glucose, an important functional domain in the sugar metabolism pathway. In addition, the two hexaploid bamboos contain more motifs involved in sugar metabolism and the above catalytic function compared to the tetraploid and diploid bamboos.

From a gene structure perspective, except for *DbUGP2* and *DbUGP3* genes, which contained 22 exons and 21 introns, all other *UGP* genes analyzed contained 21 exons and 20 introns. In comparison, previous studies have shown that the *PtrUGP* genes contained 21 exons and 20 introns [3], *AtUGP* genes contained 19 exons and 18 introns, and *StUGP* genes contained 20 exons and 19 introns [21]. Moreover, the lengths of the 18 exons in the middle of the 8 genes are the same, with minor differences in the length of the first and last 1–2 exons of each gene.

The UGP protein in potatoes has been shown to contain Lys residues and N-N/Q-S motifs that exhibit catalytic activity and substrate-binding functions [22]. Based on the distribution pattern of these residues, some scholars have speculated that in OsUGP1 protein, Lys361 was responsible for catalytic activity, while Lys257, Lys323, and Lys440 were responsible for substrate binding, including nucleotide and sugar-nucleotide binding [23]. Additionally, the first N in the motifs 162–164 (N-Q-S) and 201–203 (N-N-S) was a potential glycosylation site, which plays a crucial role in protein structural stability, functional regulation, cell recognition, and signal transduction. Moreover, previous studies on plants like *S. tuberosum* have shown that their UGP proteins share highly similar distributions of K-residues and N-N/Q-S motifs with *O. sativa* UGPase. In this study, we identified corresponding K-residues in the *D. brandisii* UGPase at positions similar to those in *O. sativa* and *S. tuberosum* and successfully located the NQS motifs and NNS motifs (except in DbUGP1 and DbUGP5) (Table S3). This is likely related to these two proteins being classified as OsUGP2-like proteins, as OsUGP2 also contains the NQS motif but lacks the NNS.

### 2.4. DbUGPs Promoters Mainly Responded to Light, MeJA, and ABA

**Figure 4:**
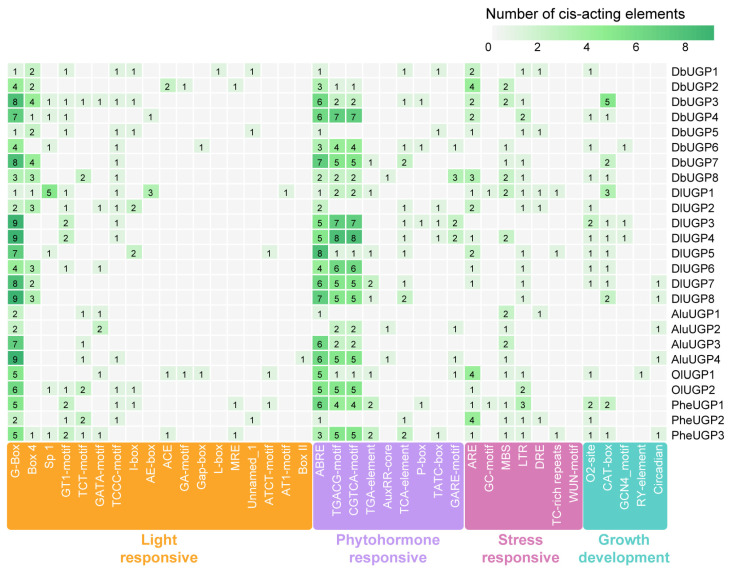
Cis-acting element analysis of the *UGP* gene family in five bamboos.

The promoter is the regulatory region of the gene, containing various cis-regulatory elements that can respond to environmental stress and regulate gene expression [24]. The cis-regulatory elements in the promoters of *DbUGPs* can be classified into four categories: light-responsive elements, hormone-responsive elements, stress-responsive elements, and growth and development-related elements (Figure 4). In the five bamboo species, almost all the genes of *D. brandisii* and *D. latiflorus* contained the four types of elements mentioned above in their promoters. Notably, the *D. brandisii* promoters have the highest number of hormone-responsive elements, with each gene containing at least two types. The most abundant are MeJA, TGACG-motif, and CGTCA-motif (42), followed by ABA, represented by ABRE (29). Compared to other bamboo species, *D. brandisii* has the most types of light-responsive elements (15), with G-box being the most prominent (36), suggesting that members of the *DbUGP* gene family may be regulated by light. The stress-responsive elements for abiotic stresses are not only present but are also the most numerous (31), with anaerobic induction (ARE and GC-motif), drought (MBS), and cold stress-related elements being particularly notable. Additionally, some secondary but equally important growth- and development-related elements were identified, such as those related to zein metabolism (O2-site), meristem expression (CAT-box), endosperm development (GCN4_motif), and circadian rhythm regulation. Except for *DbUGP2* and *DbUGP5*, every other *DbUGP* gene promoter contained at least one growth and development-related element. Among these, the CAT-box is significantly more enriched in the *DbUGP3* promoter, suggesting that *DbUGP3* is likely to be regulated by this element.

### 2.5. Prediction of Tertiary Structure and Secondary Structure Content in DbUGP Proteins

**Figure 5:**
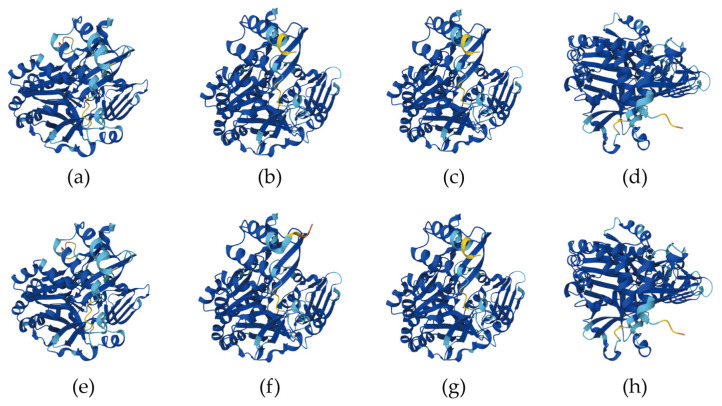
Prediction of the three-dimensional structure of DbUGP proteins. (**a**–**h**) represent the 3D structures of the DbUGP1-DbUGP8 proteins.

Using the AlphaFold3 online protein structure prediction server and the structure of *O. sativa* UGP protein as a template, we performed homologous modeling of the tertiary structure of *D. brandisii* (Figure 5). The average coverage between models was approximately 96%, with a confidence level of about 93%. DbUGP1-3 and DbUGP5-7 generally share similar structures, with subtle differences between DbUGP1 and DbUGP5 that are not statistically significant. In contrast, DbUGP4 and DbUGP8 are also highly similar but exhibit significant conformational differences when compared to the other six proteins. Additionally, we used the SOPMA tool to predict the secondary structure content of the eight DbUGP proteins (Table S4). The results showed that unordered coils (39.5%) and α-helices (36.1%) are the most abundant secondary structure elements in this family. In the tertiary model, these regions appeared as alternating flexible and regularly folded areas, providing flexibility and dynamic adaptability for catalytic activity or substrate binding, allowing efficient interaction with substrate molecules. Therefore, we analyzed the previously predicted Lys catalytic activity sites and found that they are indeed located on the unordered coil structures. Furthermore, the proportion of β-sheets (17.9%), which plays a key role in the stability, rigidity, protein core structure, mechanical strength, and support of functional regions, was also relatively high. This was complemented by a small proportion of β-turns (6.3%), which contribute flexibility and connectivity in altering protein direction, surface functional regions, and molecular binding.

### 2.6. Collinearity, Evolutionary Selection Pressure, and Replication Type of DbUGPs

**Figure 6:**
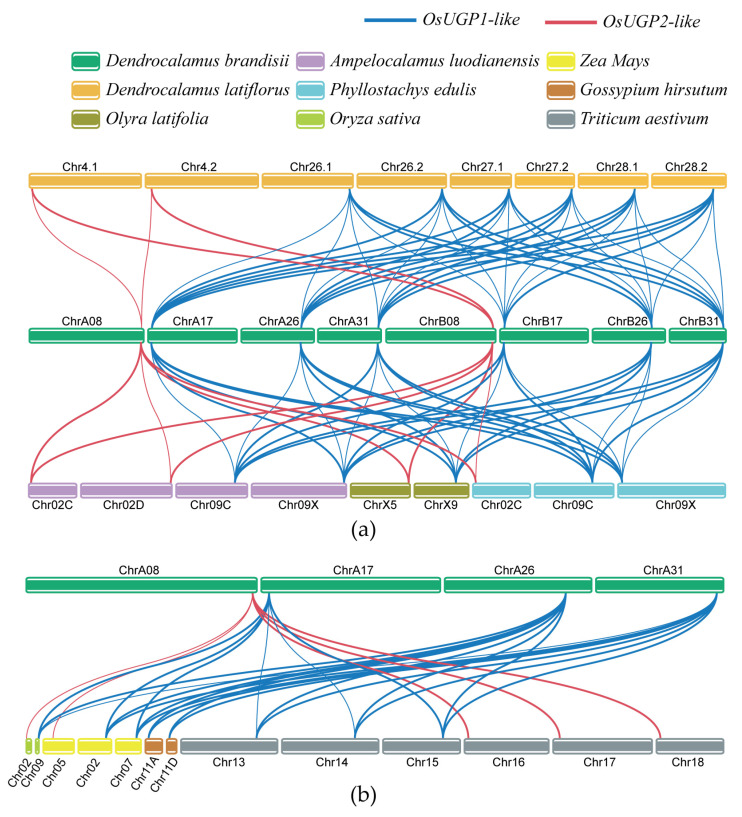
(**a**) The collinearity relationships of *UGP* genes among five bamboo species; (**b**) The collinearity relationships of the *UGP* gene family in *D. brandisii* with *O. sativa*, *Z. mays*, *G. hirsutum,* and *T. aestivum*. Blue lines represent *OsUGP1-like* genes, red lines represent *OsUGP2-like* genes, and different colored blocks represent chromosomes of different species.

Genome collinearity analysis between different species can provide clearer insights into genomic evolutionary events such as gene family expansion and chromosomal rearrangements. In this study, we performed collinearity analysis of the genome of *D. brandisii* and *O. sativa*, *Z. mays*, *T. aestivum,* and four representative bamboo species, as well as two dicotyledonous plants, *P. tomentosa* and *Gossypium hirsutum* (Figure 6). This included three model plants, seven monocotyledonous species from the same family, and two eudicot plants. As expected, *D. brandisii* showed strong collinearity with the six monocotyledonous plants within the same family (Figure 6a). All eight genes were collinear with these species, and these species shared collinear genes, indicating that the *DbUGP* gene was highly conserved in monocotyledonous plants. Here, based on the close phylogenetic relationship between *DbUGP* and *OsUGP*, we tentatively classified them into two groups: *OsUGP1-like* genes and *OsUGP2-like* genes. Each group exhibited high conservation, and the number of collinear gene pairs within each group is proportional to its own gene count. *DbUGP* had the most collinear gene pairs with *D. latiflorus*, with 40 pairs, followed by *A. luodianensis*, *O. latifolia*, and *P. edulis*, with 19, 8, and 14 pairs, respectively. Additionally, we counted the total number of collinear genes with *D. latiflorus* in three subgenomes, A, B, and C, in the order of 16, 12, and 12. In contrast, we selected two dicotyledonous species with rich fiber research backgrounds, *G. hirsutum* and *P. tomentosa*, to explore the collinearity between their *UGP* gene families and the *D. brandisii UGP* gene family. The results showed that only two pairs of *DbUGP* alleles exhibited collinearity with *G. hirsutum* genes, and no collinear genes were found with *P. tomentosa* (Figure 6b).

We calculated the selection pressure for these genes (Table S5). The effective lengths (EffectiveLen) of different gene pairs are similar. The Ka/Ks ratios for all syntenic gene pairs were less than 1, with the highest value being 0.61, and the synonymous substitution rate (pS) is generally higher than the nonsynonymous substitution rate (pN).

**Figure 7:**
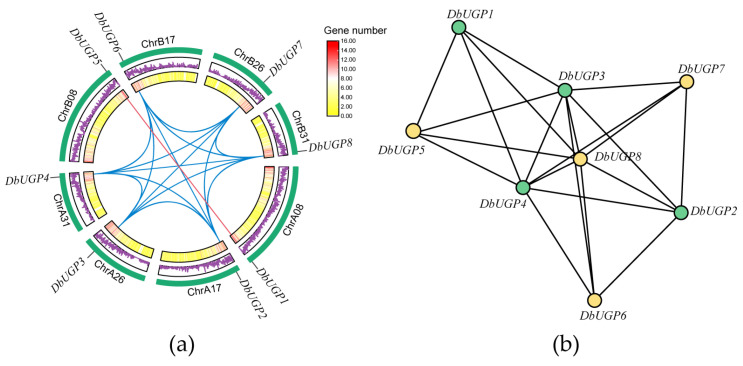
(**a**) Chromosomal location and collinearity analysis of *DbUGP* family genes; (**b**) Duplication types of the *DbUGPs*.

Furthermore, we visualized the chromosomal locations and collinearity of the *UGP* gene family within the *D. brandisii* species. Chromosomes are represented by green boxes, *OsUGP1-like* genes are connected with blue lines, and *OsUGP2-like* genes are connected with red lines. We found that *DbUGP* was evenly distributed across the four chromosomes in each subgenome, and it is evident that tandem duplication events have been excluded (Figure 7a). To further investigate the duplication events within the *DbUGP* gene family, we performed homology analysis and identified segmental duplication events involving eight members in the subgenomes (Figure 7b). The green and yellow circles represent contributions from two subgenomes, with connecting lines representing duplication relationships. Among the eight genes, there are 20 such relationships. Segmental duplication relationships occur five times between the A and B subgenomes, eight times between the A and C subgenomes, and four times between the B and C subgenomes (Table S6).

### 2.7. Transcriptome Analysis of DbUGPs in Different Organs and Culms at Different Developmental Stages

**Figure 8:**
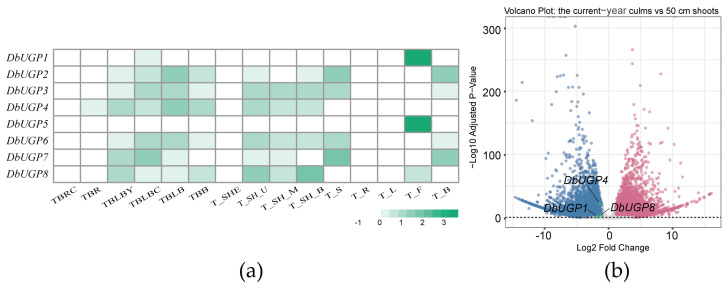
(**a**) Expression patterns of *UGP* genes in various organs or tissues of *D. brandisii*. TBRC: root primordium; TBR: root tip; TBLBY: annual bud; TBLBC: current-year bud; TBLB: bud primordium; TBB: buds at the base of the culm; T_SHE: sheath; T_SH_U/M/B: the upper/middle/basic of 50 cm tall shoots; T_S: culms; T_R: root; T_L: leaf; T_F: flowers; T_B: branch. (**b**) The gene expression trends in current-year *D. brandisii* culms compared to the 50 cm *D. brandisii* shoots. The dashed line in the figure represents padj = 0.05, with values above the line indicating padj < 0.05 and values below the line indicating padj > 0.05. Blue dots indicate genes with significantly downregulated expression, red dots indicate genes with significantly upregulated expression, and gray dots for genes with no significant change.

To elucidate the role of *DbUGPs* in the growth and development of *D. brandisii*, we performed transcriptome sequencing on different developmental stages and tissues/organs of the species. A heatmap analysis of expression patterns for eight *DbUGP* genes across 15 different tissues and organs revealed that *DbUGP1* and *DbUGP5*, previously classified as *OsUGP2-like* genes, were highly expressed specifically in flowers. In contrast, the other six genes exhibited substantial expression levels in roots, culms, leaves, and other organs (Figure 8a). This finding differed from previous studies in *O. sativa*, where *OsUGP1* was shown to predominantly regulate floral organ development. Furthermore, differential expression analysis between 50 cm tall *D. brandisii* shoots and current-year culms (Figure 8b) identified three *DbUGP* family members—*DbUGP1*, *DbUGP4*, and *DbUGP8*—as differentially expressed genes (DEGs), with significantly downregulated expression in culms compared to shoots.

### 2.8. WGCNA, PPI, and KEGG Analyses Suggested DbUGP1 and DbUGP4 Involvement in Growth, Metabolism, and Stress Adaptation

**Figure 9:**
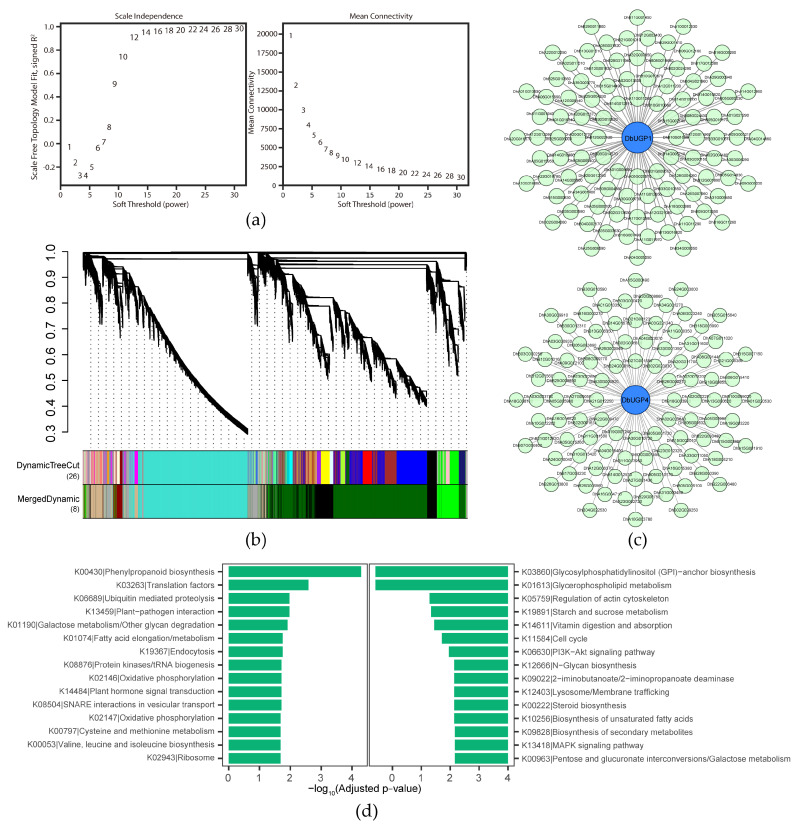
(**a**) Soft-thresholding power selection for WGCNA showing scale independence and mean connectivity. (**b**) Gene clustering dendrogram and module assignment of *DbUGP*s based on weighted gene co-expression network analysis (WGCNA). Genes are clustered into multiple co-expression modules, with each module represented by a different color. (**c**) Protein-protein interaction (PPI) networks of DbUGP1 and DbUGP4. (**d**) KEGG pathway enrichment analysis of genes co-expressed with *DbUGP1* and *DbUGP4*.

Based on Weighted Gene Co-expression Network Analysis (WGCNA), we applied a soft-threshold screening approach to classify genes into distinct co-expression modules (Figure 9a, b). Among them, *DbUGP1*, *DbUGP4*, and *DbUGP8* were clustered in the dark-green module; *DbUGP2* and *DbUGP7* were assigned to the turquoise module; while *DbUGP6* was classified into the tan module. Genes within the same module are likely to share similar functions or participate in common metabolic pathways. In contrast, the gray module comprises genes that could not be categorized into any specific module, thus lacking biological significance.

Building upon the previous analyses, we selected two genes of particular interest from the *OsUGP1-like* and *OsUGP2-like* genes in *D. brandisii*, namely *DbUGP1* and *DbUGP4*, for further investigation. A protein-protein interaction (PPI) network was constructed for each of these genes, incorporating 100 interacting proteins based on connectivity ranking (Figure 9c). Subsequently, KEGG (Kyoto Encyclopedia of Genes and Genomes) enrichment analysis was performed on 100 genes co-expressed with *DbUGP1* and *DbUGP4*, revealing both similarities and differences (Figure 9d). The similarities include: 1) *DbUGP1*-associated genes were enriched in “galactose metabolism/other glycoside degradation” (K01190), while *DbUGP4*-associated genes were enriched in “pentose and glucuronic acid interconversion/galactose metabolism” (K00963), both pointing to the central role of UDP-glucose in carbohydrate metabolism; 2) *DbUGP1* was primarily associated with “phenylpropanoid biosynthesis” (K00430), while *DbUGP4* showed broader involvement in “secondary metabolites biosynthesis” (K09828), suggesting that both *UGP* genes are closely linked to plant secondary metabolism and biosynthesis processes, such as cell wall formation. The differences lie in that *DbUGP4* is more strongly associated with cell cycle control and sugar/starch metabolism, while *DbUGP1* also exhibited enrichment of genes related to fundamental cellular processes, such as protein synthesis/degradation, energy supply (oxidative phosphorylation), endocytosis, and vesicular transport. This also corroborates the differential conformation observed in DbUGP1 (a) and DbUGP4 (d) protein structures in Figure 5. These findings provide strong evidence that *DbUGP1* and *DbUGP4* may be involved in carbohydrate metabolism, cell wall biosynthesis, and other related processes.

### 2.9. qRT-PCR Validation of Transcriptome Data from Various Tissues and Organs of D. brandisii

**Figure 10:**
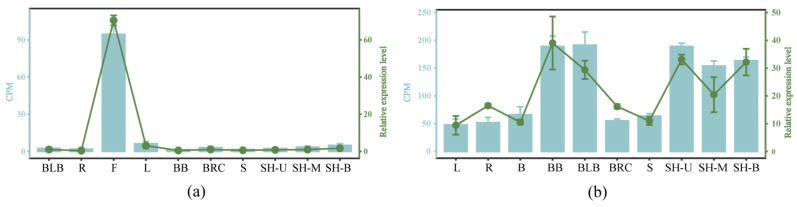
Validate the transcriptome data by qRT-PCR. (**a**) qRT-PCR validation results of *DbUGP1*; (**b**) qRT-PCR validation results of *DbUGP4.* CPM: Counts per million, refers to the normalized gene expression level provided by RNA sequencing data; Relative expression level: The qRT-PCR experimental results of this gene in different tissues and organs. L: leaf; R: root; B: branch; F: flower; BB: buds at the base of the culm; BLB: bud primordium; BRC: root primordium; S: culm; SH-U/M/B: the upper/middle/basic of 50 cm tall shoots.

To further validate the RNA-seq data, a qRT-PCR analysis was conducted on selected members of the *DbUGP* gene family in the same organs and tissues used for RNA-seq. In this study, *DbUGP1* and *DbUGP4* were chosen for quantitative analysis based on the criterion that their RNA-seq data exhibited significant fluctuations across different samples, allowing for a clearer observation of whether the trends in gene expression are consistent. The reference gene used was *EF-1-α-2* [25], which is stably expressed across all tissues and organs and has high primer specificity. The visualization of the qRT-PCR results confirmed the accuracy of the transcriptome data (Figure 10), showing that the expression patterns of *DbUGPs* in various organs and tissues were generally consistent with those observed in the RNA-seq data.

## 3. Discussion

In recent decades, UGP protein members have gradually been discovered across various species and are highly conserved, representing important rate-limiting enzymes involved in the entire life cycle of plants. During plant growth, they participate in a series of carbohydrate metabolic pathways across all tissues, including the reversible catalysis of UTP and Glc-1-P to form UDP-Glc and pyrophosphate. The catalytic direction and efficiency vary in different tissues and organs, thereby affecting the distribution of various types of sugars [26]. However, a systematic identification and functional annotation of this gene family in *D. brandisii*, known for its unique and complex bamboo shoot developmental characteristics, is currently lacking. Research has shown that most plants, such as *A. thaliana* [2], *O. sativa* [23,27], *S. tuberosum* [28], *Musa nana* [29], *Astragalus membranaceus* [30], *Cucumis melo* [31], *Hordeum vulgare* [32], and *P. tomentosa* [3] possess two highly similar *UGP-A* genes (*UGP1*, *UGP2*) encoding UGP-A proteins. In addition, there is usually a single *UGP-B* gene encoding UGP-B proteins [23]. According to the literature review, numerous studies have focused on the *UGP-A* gene, possibly because the UGP-B protein shares low or no homology with UGP-A. Additionally, *UGP-B* is typically reported to encode a smaller chloroplastic isoform, with its function seemingly restricted to the synthesis of UDP-sulfoquinovose [2,3]. In this study, we performed a comprehensive genome-wide identification of the *UGP-A* gene in *D. brandisii* and identified eight *DbUGP* genes distributed across eight chromosomes. The sequences of the encoded DbUGP proteins all contain the typical UDPGP functional domain, consistent with the typical structure of UGP proteins in various plant species [33,34].

The subcellular localization of UGP-A and UGP-B in plants has yielded differing conclusions. Immunolabeling experiments have shown that UGP-A is primarily localized in the cytoplasm of various plants such as *O. sativa*, *A. thaliana*, *Nicotiana tabacum*, and *S. tuberosum*. However, it has also been observed at certain levels in plastids, Golgi apparatus, and microsomes [35,36,37]. On the other hand, UGP-B proteins are specifically localized in the chloroplasts, where they play a crucial role as key enzymes in the initial steps of galactolipid biosynthesis, unique to *A. thaliana* chloroplasts [38]. Our predictions suggest a cytoplasmic localization (Table S1). In fact, we have already determined the subcellular localization of DbUGP4 in *D. brandisii* seedling leaf protoplasts, and the result shows chloroplast localization. Additionally, in *N. tabacum* leaves, we found that DbUGP1 localizes to the Golgi apparatus (unpublished).

Although the *UGP* gene family is a relatively small gene family, the number of its members is generally proportional to the ploidy level of the plant. Phylogenetic analysis reveals that *UGP* genes from several dicot species form a distinct clade and exhibit significant evolutionary divergence from monocot *UGP* genes (Figure 2). *DbUGPs*, as members of the bamboo subfamily (Poaceae, Monocotyledon), cluster closely with the two *O. sativa UGP* genes (*OsUGP1* and *OsUGP2*) and show a closer genetic relationship to *OsUGPs* than to *AtUGPs*. (Figure 2). Based on the number of family members, it can be inferred that *D. brandisii* has experienced *UGP* gene family expansion compared to herbaceous bamboos like *O. latifolia* or relatively low-ploidy bamboo species such as *P. edulis* and *A. luodianensis*, especially in the *OsUGP1-like* genes. This suggests that the *OsUGP1-like* genes may be one of the key factors contributing to the efficient carbon metabolism and superior taste quality of edible shoots in *D. brandisii*. Finally, except for the two allelic genes, *DbUGP4* and *DbUGP8*, most other allelic genes were more homologous to a gene from *D. latiflorus* than to other *D. brandisii* alleles. This not only suggests that the *UGP* in these two bamboo species are very closely related but also implies that *DbUGP4* and *DbUGP8* may have evolved unique roles within the gene family; they may be potentially important genes whose roles warrant further investigation.

The analysis of the *DbUGPs* structure and motifs shows high conservation (Figure 3). However, the separation into different phylogenetic branches may result from subtle variations in non-motif regions, gene duplication, selective pressures, and sequence construction methods. This highlights the coexistence of evolutionary diversity and functional conservation. Overall, not all conserved motifs have a clear, defined function (Table S2). While some motifs are highly conserved in sequence, they may simply serve as structural stabilizers or evolutionary remnants rather than directly participating in catalysis or functional regulation. It is also possible that some motifs, such as motif 9 and motif 10, possess very specific functions that have yet to be discovered. As research techniques (e.g., structural biology, proteomics) advance, more novel functions of these motifs may be revealed in the future. In addition to the previous summary of the exon characteristics of *DbUGPs*, the often-overlooked introns are also crucial in plant evolution. Non-coding regions are essential for regulating mRNA stability and gene expression [39]. In *D. brandisii*, the number of introns in certain genes reaches up to 21, which is higher than that found in other species studied in this research. This may suggest the advanced evolutionary status of *D. brandisii* in plant evolution. DbUGPs share highly consistent catalytic activity and substrate-binding sites with the previously characterized OsUGP and StUGP, suggesting that they may perform similar functions (Table S3). However, DbUGP1 and DbUGP5 contain the NQS motif but lack the NNS motif, which corresponds to a similar situation in OsUGP2 [23]. The impact of missing a glycosylation site on the enzyme’s stability and overall functionality remains to be studied, and it is an intriguing research topic to investigate whether this absence is one of the reasons why these two genes are more specifically expressed in flowers compared to other *DbUGPs*.

All *DbUGPs* show highly conserved collinearity with *UGPs* from monocots, particularly with those from species within the Bambusoideae subfamily of the Poaceae, indicating the closest phylogenetic relationship [40] (Figure 6). In contrast, the collinearity with *UGPs* from dicot species is very limited or nearly nonexistent (Figure 6b). This suggests that *D. brandisii* and *G. hirsutum* may have preserved key genomic segments inherited from their common ancestor. However, due to the independent genome recombination and specialization in dicots, significant gene loss or reshuffling occurred, resulting in reduced collinearity with monocots and a more distant evolutionary relationship. Furthermore, in the A subgenome, the proportion of *DbUGPs* forming orthologous pairs with *UGPs* from other species is higher than that in subgenomes B and C, suggesting that *DbUGPs* in the A subgenome are more conserved. Previous studies have shown that hexaploid woody bamboos have complex origins and have undergone intricate reticulate evolution [41]. We speculate that this may explain the significant variation in the number and homology of *DbUGPs* among different subgenomes and species. The Ka/Ks ratio analysis of the collinear gene pairs between *D. brandisii* and seven monocot species indicates that the synonymous substitution rate greatly exceeds the nonsynonymous substitution rate, ensuring the catalytic activity and specific functions of the UDPGP functional domain remain highly conserved (Table S5). This generally suggests that these genes have undergone strong purifying selection during their genetic evolution. At the same time, gene families are primarily generated through six different mechanisms: whole-genome duplication, tandem duplication, segmental duplication, retrotransposon insertion, exon duplication, and rearrangement [42]. Our gene duplication analysis reveals that segmental replication, rather than tandem duplication, is the primary mechanism behind the expansion of the *DbUGP* gene family during evolution (Figure 6). In this study, segmental duplications were relatively concentrated between the A and C subgenomes (Table S6), which may suggest that these subgenomes are more closely related in evolution and have undergone a greater number of segmental duplication events together. In contrast, we speculate that the B subgenome may have originated from a different ancestor or experienced stronger genome rearrangement or selection pressure. Moreover, the repeated copies of *DbUGPs* likely facilitated the expansion of the gene family and may have led to functional redundancy [43,44]. This redundancy can compensate for gene functions under specific conditions, thereby maintaining the normal growth of *D. brandisii*, particularly in the functions regulated by *OsUGP1-like* genes.

The modeling results of the *DbUGP* gene family indicate that six proteins share a consistent structure, while the other two exhibit a distinct yet internally consistent structural pattern. This finding suggests that the *DbUGP* gene family may have undergone a certain degree of functional divergence. Although these proteins still belong to the same evolutionary family and likely retain similar core functions, some members may have undergone structural adaptations to accommodate specific biological functions. This observation does not contradict the previous analysis based on primary sequence similarity, as tertiary protein structures are also influenced by factors such as secondary structure formation rules, solvent accessibility, and hydrogen bonding. Even with high sequence similarity, variations in protein folding patterns may result in the formation of two distinct stable conformations. (Figure 5). The catalytically active sites are primarily located in the disordered coil regions of the secondary structure, along with stable and rigid β-sheets (Table S4). A few β-turns contribute flexibility and connectivity, playing an important role in altering protein orientation, surface functionality, and molecular binding. Overall, these characteristics confer stability, regularity, and flexibility to DbUGP proteins, enabling them to perform efficiently in complex biological processes.

Transcriptomic data obtained from different developmental stages of *D. brandisii* shoots and mature bamboo indicate that *DbUGP1* and *DbUGP5* (*OsUGP2-like* genes) are highly expressed in floral organs, suggesting their specific roles in flower development. In contrast, the other six genes (*OsUGP1-like* genes) are predominantly expressed in tissues such as rhizomes, roots, and leaves (Figure 8a). This expression pattern aligns with previous research in *O. sativa*, where two *UGP* genes are known to dominate flower development and other tissue development, respectively. Several *DbUGPs* (such as *DbUGP1*, *DbUGP4*, and *DbUGP8*) show a significant downregulation in expression as the 50 cm bamboo shoot develops into the current-year bamboo (Figure 8b). Additionally, the promoter cis-elements analysis revealed a high presence of light-responsive and hormone-responsive elements, suggesting that the downregulation of *DbUGPs* might be linked to the developmental transition. During the bamboo shoot developmental stage, rapid cell division and growth require a substantial supply of sugars and hormonal signals. However, the current-year bamboo, with its stable survival strategy and reduced need for light and hormones [45], shows decreased *DbUGP* expression as cellular maturation and lignification near completion. Therefore, through WGCNA, PPI, and KEGG pathway enrichment analysis, we preliminarily explored the potential role of *DbUGP1* and *DbUGP4* in carbohydrate metabolism. These genes appear to regulate UDP-glucose levels (K01190, K00963), thereby influencing cell wall formation, energy storage, and sugar signaling. *DbUGP4* involvement in starch and sucrose metabolism (K19891) suggests a role in plant growth and stress adaptation. Additionally, the enrichment of *DbUGP4* in lipid metabolism (K10256, K09828) indicates a potential function in modulating membrane fluidity and brassinosteroid signaling, which may regulate cell division, elongation, and culm development. The enrichment of phenylpropanoid biosynthesis (K00430), N-glycosylation (K12666), and GPI-anchored protein biosynthesis (K03860) suggests a possible involvement in cell wall biosynthesis. Furthermore, their association with cell cycle regulation (K11584), cytoskeletal dynamics (K05759), and key signaling pathways (K08876, K06630, K13418) indicates their broad involvement in plant growth, development, and environmental adaptation. However, the differences in the enriched pathways of the co-expressed genes of *DbUGP1* and *DbUGP4*, coupled with their distinct protein conformations and subcellular localizations, suggest that they may perform partially distinct or functionally specialized roles during plant growth and development. In conclusion, these findings are relatively consistent with our expected functional roles of *DbUGPs*. In summary, these findings are relatively consistent with our expected functional roles of *DbUGPs*, reflecting the reliability of our analysis to some extent. This study provides meaningful insights that may serve as a valuable reference for future research on the molecular mechanisms of sugar synthesis and metabolic regulation in *D. brandisii*.

## 4. Materials and Methods

### 4.1. Identification and Analysis of UGP Gene Families in D. brandisii

The high-accuracy genome and transcriptome data of *D. brandisii* were sequenced and published in previous studies by our research team [19] and can be downloaded from the Figshare platform with DOI 10.6084/m9.figshare.24455197, as well as from the NCBI Sequence Read Archive (SRA) under BioProject PRJNA885281. Genome data for other bamboo species were retrieved from BambooBase, a comprehensive resource for bamboo genomics and systematics (https://bamboo.genobank.org/index.html, accessed on 16 May 2024). Gene sequences for other species, such as *O. sativa*, *A. thaliana*, and others, were obtained from NCBI (https://www.ncbi.nlm.nih.gov/, accessed on 16 May 2024). The protein sequences of UGPs were aligned with AtUGPs and OsUGPs using the BLASTp program (Version 2.15.0) from the National Center for Biotechnology Information (NCBI). The HMM profile PF01704 from Pfam was used to identify high-quality protein sequences with an e-value lower than 1e^−100^. The integrity of the identified UGP protein sequences was further verified using InterPro (http://www.ebi.ac.uk/interpro/search/sequence/, accessed on 18 May 2024) [46] and SMART (http://smart.Embl-heidelberg.de/, accessed on 18 May 2024) [47]. The homology of *UGPs* from *P. edulis*, *A. luodianensis*, *O. latifolia*, *T. aestivum,* and other species was identified in the same manner.

### 4.2. Physical and Chemical Properties of DbUGPs

We analyzed the physicochemical properties, transmembrane domains, signal peptides, and subcellular localization of *DbUGPs* using ExPASy (https://web.expasy.org/protparam/, accessed on 26 May 2024) [48], DeepTMHMM (https://dtu.biolib.com/DeepTMHMM/, accessed on 26 May 2024) [49], SignalP 6.0 (https://services.healthtech.dtu.dk/services/SignalP-6.0/, accessed on 26 May 2024) [50], and WoLF PSORT (https://wolfpsort.hgc.jp/, accessed on 26 May 2024) [51]. We used MEME (Version 5.5.7, https://memesuite.org/meme/doc/meme.html, accessed on 26 May 2024) [52] to identify conserved motifs in *DbUGPs*, with the following parameters: maximum e-value = 1e^−5^, number of motifs = 10, minimum motif length = 29, and maximum motif length = 50. Subsequently, we used TBtools (Version 2.225) [53] to generate conserved domain sequence logos and gene structure views for *DbUGPs*. We extracted 2000 bp sequences upstream of the start codon (ATG) for each *DbUGP* and predicted the cis-elements using PlantCARE (http://bioinformatics.psb.ugent.be/webtools/plantcare/html/, accessed on 28 May 2024) [54]. We used the AlphaFold3 online protein structure prediction server (https://alphafold.ebi.ac.uk/search/sequence/, accessed on 1 May 2025) [55] to perform homology modeling of the tertiary structure of DbUGP proteins, using the high-confidence crystal structure of OsUGP protein as a template. Additionally, we used the SOPMA tool (https://npsa-prabi.ibcp.fr/cgi-bin/npsa_automat.pl, accessed on 8 January 2025) [56] to assist in predicting the secondary structure content of the eight DbUGP proteins.

### 4.3. Phylogenetic, Collinearity, Evolutionary Selection Pressure, and Duplication Analysis of DbUGPs

We performed multiple sequence alignments of these genes using Muscle 3.8 [57] and automatically trimmed the alignment results using trimAL 1.4 [58]. A maximum likelihood (ML) tree was constructed using FastTree 2.1.10 [59], and the phylogenetic tree was visualized using ChipLot [20]. To provide a clearer representation of the phylogenetic relationship among *UGP* genes, we manually removed branches without *DbUGP*. We conducted inter-species collinearity analysis using MSCanX [60], which also provided information on segmental duplications. The results were then visualized using R. Additionally, chromosomal localization, chromosomal density, and within-species collinearity analysis were visualized using the Advanced Circos module in TBtools (Version 2.225). Finally, we estimated the non-synonymous (Ka) and synonymous (Ks) substitution values of duplicated *DbUGP* gene pairs using TBtools (Version 2.225).

### 4.4. Analysis of Gene Expression Patterns

To analyze the expression differences of *DbUGPs* across different tissues or organs, we created a heatmap using transcriptome data from the aforementioned plant materials. Prior to generating the heatmap using R Studio (Version 4.4.2), expression values for the same gene across different organs were standardized using the Z-score normalization method. To compare the upregulation and downregulation of gene expression between the current-year mature culms and shoots of *D. brandisii*, we performed differential expression analysis using the DESeq2 package in R [61]. Genes were selected based on adjusted p-values (padj < 0.05) and log2 fold changes, with log2FoldChange > 1 defined as upregulated genes and log2FoldChange < −1 defined as downregulated genes. Volcano plots were generated based on these criteria.

In the WGCNA analysis, genes with an average expression level below 0.5 and low expression variation (standard deviation ≤ 0.1) were filtered out. A total of 38,858 genes and 12 samples remained, with a module merging threshold of 0.35. The power value was set from 1 to 30, and the correlation coefficients and average connectivity for each corresponding network were calculated. The power value of 12 was selected based on the analysis results, and a weighted gene co-expression network model was constructed. Genes were divided into 8 modules, and a co-expression network was constructed based on gene co-expression correlations, with the target genes as the central focus of the network. The KEGG pathway annotation in this study integrated multiple public protein and domain databases, primarily utilizing NCBI BLASTp (Version 2.15.0) to perform homologous alignment of *D. brandisii* genes in the KEGG plant database (http://www.genome.jp/kegg/, accessed on 6 January 2025), with the best alignment results used for annotation. Data analysis was conducted using the Hypergeometric test to calculate the enrichment significance *p*-values of proteins in various KEGG pathways within the network. To reduce the false positive rate, multiple testing correction of *p*-values was performed using the False Discovery Rate (FDR) method, yielding adjusted *p*-values, which were then ranked accordingly.

### 4.5. qRT-PCR Validation of the Accuracy of Transcriptome Data

Healthy plant organs or tissues (0.5 g) from the same locations as the transcriptome sequencing were taken from a −80 °C ultra-low temperature freezer and immediately frozen in liquid nitrogen to prevent RNA degradation. The samples were ground into a fine powder using a pre-cooled mortar and pestle, and RNA was extracted using the Trizol method. The RNA was then reverse-transcribed into cDNA using the gDNA Eraser (TaKaRa) kit. Primers were designed using Primer 5 (Table S7), and primer specificity was verified using the NCBI online tool (primer-blast). The reference gene used was *EF-1-α-2* [25]. A 20 µL reaction mixture was prepared (SYBR Master Mix 10 µL, upstream primer 3 µL, downstream primer 3 µL, template cDNA 3 µL, and ddH_2_O 1 µL) and subjected to qRT-PCR analysis on a LightCycler 480 real-time system (Roche, Rotkreuz, Switzerland). Three biological replicates were performed to calculate the relative expression of the target gene, and expression data were analyzed using the 2^−ΔΔCT^ method [62].

## 5. Conclusions

In this study, eight members of the gene family harboring the UDPGP domain were identified and classified into two subfamilies. These members exhibited high conservation in gene structure and motif composition, although their protein tertiary structures diverged into two distinct conformations. Segmental duplication events were detected within this gene family, while no tandem duplication was observed. Analysis of cis-acting elements in the promoter regions of *DbUGPs* revealed responsiveness to light, methyl jasmonate (MeJA), and abscisic acid (ABA) treatments. RNA-seq expression profiling demonstrated that *DbUGP1* and *DbUGP5* were highly expressed in floral organs, suggesting potential roles in flower development, while the remaining six genes displayed ubiquitous expression across multiple tissues. Notably, a significant downregulation trend was observed in the expression levels of *UGP* family genes during the developmental transition from shoots to mature culms, particularly for *DbUGP1*, *DbUGP4*, and *DbUGP8*. Further investigations involving metabolite profiling or functional validation of these genes are recommended. This study provides comprehensive functional predictions for *DbUGPs* and identifies candidate genes for elucidating sugar synthesis/metabolism and cell wall development in *D. brandisii*.

## Data Availability

The original contributions presented in this study are included in the article/Supplementary Materials. Further inquiries can be directed to the corresponding author.

## Supplementary Materials

The following supporting information can be downloaded at: https://www.mdpi.com/article/10.3390/plants14101458/s1. Table S1: Basic information for the *Dendrocalamus brandisii UGP* gene family members. Table S2: The detailed information of conserved motifs in *DbUGPs.* Table S3: Prediction of Lys catalytic activity and N-N/Q-S substrate-binding sites in DbUGPs proteins. Table S4: Prediction of secondary structure composition in DbUGPs proteins. Table S5: Ka/Ks ratios for paralogous gene pairs. Table S6: Subgenomes and duplication type for gene pairs of *DbUGPs*. Table S7: The qRT-PCR primer sequences for the reference gene, *DbUGP1* and *DbUGP4*.
